# ﻿Primary prevention implantable cardioverter-defibrillator use in non-ischemic dilated cardiomyopathy based on arrhythmic risk stratification and left ventricular reverse remodeling prediction﻿

**DOI:** 10.1007/s10741-022-10246-6

**Published:** 2022-05-19

**Authors:** Ahmed Muhammed, Mohamed Abdelazeem, Mohamed Gamaleldin Elewa, Mohamed Sharief, Ahmed Ammar

**Affiliations:** 1 Cardiology Department, Faculty of Medicine, Ain Shams University, Cairo, Egypt; 2 Department of Medicine, St. Elizabeth’s Medical Center, Boston, MA USA; 3 Department of Medicine, Tufts University School of Medicine, Boston, MA USA; 4 Cardiology Department, Mansoura University Hospital, El Mansoura, Egypt; 5 Lancashire Teaching Hospitals NHS Foundation Trust, Preston, UK; 6 Cardiology Department, Worcestershire Acute Hospitals NHS Trust, Worcester, UK

**Keywords:** Non-ischemic dilated cardiomyopathy, Sudden cardiac death, Implantable cardioverter defibrillator

## Abstract

Sudden cardiac death (SCD) and significant ventricular arrhythmias in patients with dilated cardiomyopathy (DCM) have been markedly reduced over the last couple of decades as a result of the advances in pharmacological and non-pharmacological treatment. Primary prevention implantable cardioverter-defibrillator (ICD) plays an important role in the treatment of patients at risk of SCD caused by ventricular arrhythmias. However, the arrhythmic risk stratification in patients with DCM remains extremely challenging, and the decision for primary prevention ICD implantation based on left ventricular ejection fraction (LVEF) solely appears to be insufficient. This review provides an update on current evidence for primary prevention ICD implantation, arrhythmic risk stratification, and left ventricular reverse remodeling (LVRR) prediction in patients with DCM in addition to most recent guideline recommendations for primary prevention ICD implantation in DCM patients and a proposed multiparametric algorithm based on arrhythmic risk stratification and left ventricular reverse remodeling (LVRR) prediction to better identify patients who are likely to benefit from primary prevention ICD.

## Introduction

DCM is defined as left ventricular dilatation and systolic dysfunction in the absence of abnormal loading conditions or coronary artery disease (CAD) sufficient to cause global systolic impairment [1].

DCM presents in people of all ages and ethnicities. In adults, it is more common in men than in women, with an overall prevalence up to 1 in 2500 in the general population [2, 3]. Genetic mutations involving genes that encode cytoskeletal, sarcomere, and nuclear envelope proteins account for up to 35% of cases.

Despite advances in pharmacological and non-pharmacological therapies of heart failure, the mortality rates remain high in patients with DCM with SCD accounting for up to 35% of all deaths among patients with DCM [4].

The LVEF is an easily measurable, quantifiable marker of SCD risk with a well-established relationship between worsening LVEF and increased risk of arrhythmic mortality. Although a statistically significant benefit is linked to ICD therapy in appropriately designed clinical trials that use LVEF as the main criterion for device selection, however, LVEF performs relatively poorly when used solely in predicting the likelihood of ICD benefit. In addition, the negative results of the DANISH (Danish study to assess the efficacy of ICDs in patients with non-ischemic systolic heart failure on mortality) trial emphasized the fact that the current criteria for primary prevention ICD implantation in DCM are not specifically selecting the highest risk population and hence the need for different way for arrhythmic risk stratification [5].

## Review of the current evidence

The role of primary prevention ICD in patients with ischemic cardiomyopathy (ICM) is well-established. The Multicentre Automatic Defibrillator Implantation Trial II (MADIT II) depicted a survival benefit from prophylactic ICD over 3 years of follow-up [6]. In the Multicenter Unsustained Tachycardia Trial (MUSTT), patients with ischemic left ventricular dysfunction and coronary artery disease with non-sustained ventricular tachycardia underwent electrophysiological study. Patients with inducible ventricular tachycardia were randomized to receive either anti-arrhythmic drugs or no treatment. ICD was implanted in patients with failure of anti-arrhythmic drug therapy to suppress inducible VT during serial EP testing. ICD significantly reduced total mortality and arrhythmic death. Anti-arrythmic drug therapy failed to reduce both outcomes [7].

Multiple randomized controlled trials (RCTs) have addressed the topic of prophylactic ICD implantation in NICM patients. In 2005, the Sudden Cardiac Death in Heart Failure Trial (SCD-HeFT) demonstrated a 23% reduction in overall mortality in heart failure with reduced ejection fraction (HFrEF) patients with single-chamber ICD as compared to each of placebo and amiodarone. In this trial, 48% of patients had NICM; however, none of the included patients received CRT and differentiation between ICM and NICM which was solely based on history [8].

The Cardiomyopathy Trial (CAT) included 104 patients with dilated cardiomyopathy who were randomized to either a prophylactic ICD or medical treatment only and after a follow-up period of 4 years; cumulative survival was not different between the 2 groups [9]. In the Defibrillators In Non-Ischemic Cardiomyopathy Treatment (DEFINITE) trial, 458 NICM patients with asymptomatic premature ventricular contractions (PVCs) or non-sustained ventricular tachycardia were randomized to medical therapy plus ICD versus medical therapy alone and the ICD group showed a significant reduction in arrhythmic sudden cardiac death, with non-significant reduction in all-cause mortality [10]. The COMPANION trial compared medical treatment versus CRT-Pacemaker (CRT-P) versus CRT-D in terms of total mortality in patients with NICM. CRT proved to reduce overall mortality, but CRT-D was non-superior to CRT-P in this regards [11].

In 2016, the DANISH trial, largest trial addressing this topic, was published including 1116 symptomatic NICM patients followed up for a mean period of 67.6 months. The DANISH trial showed no significant difference between the ICD group and optimal medical therapy (OMT) group as regards the primary outcome of all-cause mortality. However, the risk of SCD was almost halved in the ICD group [12]. Age was an effect modifier with less benefit at older age and potential harm was found in patients older than 69 years. In addition, a subgroup analysis of the DANISH trial showed a reduction of all-cause mortality with ICDs implanted at an age of 70 years or younger which could be explained on the basis that the older patients are more likely to die because of non-cardiac or non-sudden cardiac death causes as compared to younger patients [13]. A meta-analysis of 5 RCTs (CAT, AMIOVIRT, SCD-HeFT, DEFINITE, and DANISH) showed a significant reduction in overall mortality and SCD with ICD compared to medical treatment alone with the benefit more pronounced in the under 60 age population [14].

A possible explanation for the discrepancy between CRT-D benefits in ICM as compared to NICM is that ICM patients are more liable to ventricular arrhythmias. This difference is boostered by the postulated better response of NICM patients to CRT implantation with the benefited LV reverse remodeling, hence reducing the risk of arrhythmic sudden death [15]. Contrary to this, a retrospective cohort study showed higher mortality rates in NICM patients receiving CRT-P as compared to CRT-D for primary prevention. However, this effect was abolished in older age groups > 75 years old [16]. Finally, the decision to implant a CRT with or without defibrillator is still debatable and should be individual-centered after proper arrhythmic risk assessment.

The role of prophylactic ICD in the asymptomatic division of heart failure patient population has also not been adequately investigated. Only two of the landmark RCTs, AMIOVIRT and DEFINITE, included asymptomatic heart failure patients (NYHA class I), representing 18% and 25.3% of the study populations, respectively [10], and more robust body of evidence is needed before drawing solid conclusions and recommendations as regards to this abandoned subgroup [17]. NYHA class IV patients are even more abandoned in this regard and only the DANISH trial included one patient in each arm [12] and hence valuable evidence in this field is scarce and needs further enrichment by dedicated prospective trials.

The randomized control trials of primary prevention ICD in DCM patients are summarized in Table 1.

**Table 1:** Summary of randomized control trials of primary prevention ICD in DCM patients

**RCT**	**CAT** [9]	**AMIOVIRT** [18]	**DEFINITE** [10]	**SCD-HeFT** [8]	**DANISH** [12]
**Year published**	2002	2003	2004	2005	2016
**Participants** (*n*)	104	103	458	2521	1116
**Arms**	ICD vs medical therapy	ICD vs amiodarone	ICD vs medical therapy	ICD vs amiodarone vs placebo	ICD + standard care vs standard care (including CRT)
**Inclusion criteria**	Symptomatic DCM ≤ 9 months, EF ≤ 35%, NYHA II or III	EF ≤ 35%, NYHA I, II, or III, asymptomatic NSVT	EF ≤ 35%, NYHA I, II, or III, NSVT or PVCs	EF ≤ 35%, NYHA II or III	EF ≤ 35%, NYHA II or III (or IV if CRT is planned), NT-pro BNP > 200 pg/ml
**Enrolment period**	1991–1997	1996–2000	1998–2002	1997–2001	2008–2014
**Follow-up (months)**	66	24	29	45.5	67.6
**Age (years)**	50/54	51/52	229/229	60/60	64/63
**Baseline medication (%)**					
**ACEI/ARB**	94/98	NR	NR	94/98	96/97
**Beta blockers**	4/4	NR	NR	69/69	92/92
**MRA**	NR	NR	NR	NR	59/57
**Medication at termination (%)**					
**ACEI/ARB**	94/98	90/81	97/96	86/88	99 of total
**Beta blocker**	NR	53/50	86/84	82/79	98 of total
**MRA**	NR	20/19	4/7	NR	79 of total
**Primary outcome**	Death from any cause	Death from any cause	Death from any cause	Death from any cause	Death from any cause
**Number with primary outcome of total**	13 of 50/17 of 54	7 of 52/6 of 51	28 of 229/40 of 229	71 of 424/95 of 417	58 of 234/65 of 237
***P*** **value for 1ry outcome**	0.554	0.8	0.08	0.06	0.28

*ACEI* angiotensin converting enzyme inhibitor, *AMIOVIRT* amiodarone versus implantable cardioverter-defibrillator randomized trial, *ARB* angiotensin receptor blocker, *EF* ejection fraction, *MRA* mineralocorticoid receptor antagonist, *NSVT* non-sustained ventricular tachycardia, *NT-pro BNP* N-terminal pro brain natriuretic peptide, *NYHA* New York Heart Association

## Arrhythmic risk stratification and LV reverse remodeling

### Arrhythmic risk stratification

Previous trials assessing role of primary prevention ICD therapy in DCM patients have all utilized ejection fraction and NYHA classification as part of their inclusion criteria [8–10, 18] (Table 1). However, those two parameters are highly operator dependent. Furthermore, the relatively low incidence of arrhythmic events in randomized clinical trials on DCM patients makes it crucial to properly risk stratify this heterogeneous population of patients with DCM in order to identify patients who will benefit from primary prevention ICD implantation.

### Arrhythmic risk stratification parameters (Fig. 1)

**Fig. 1:**
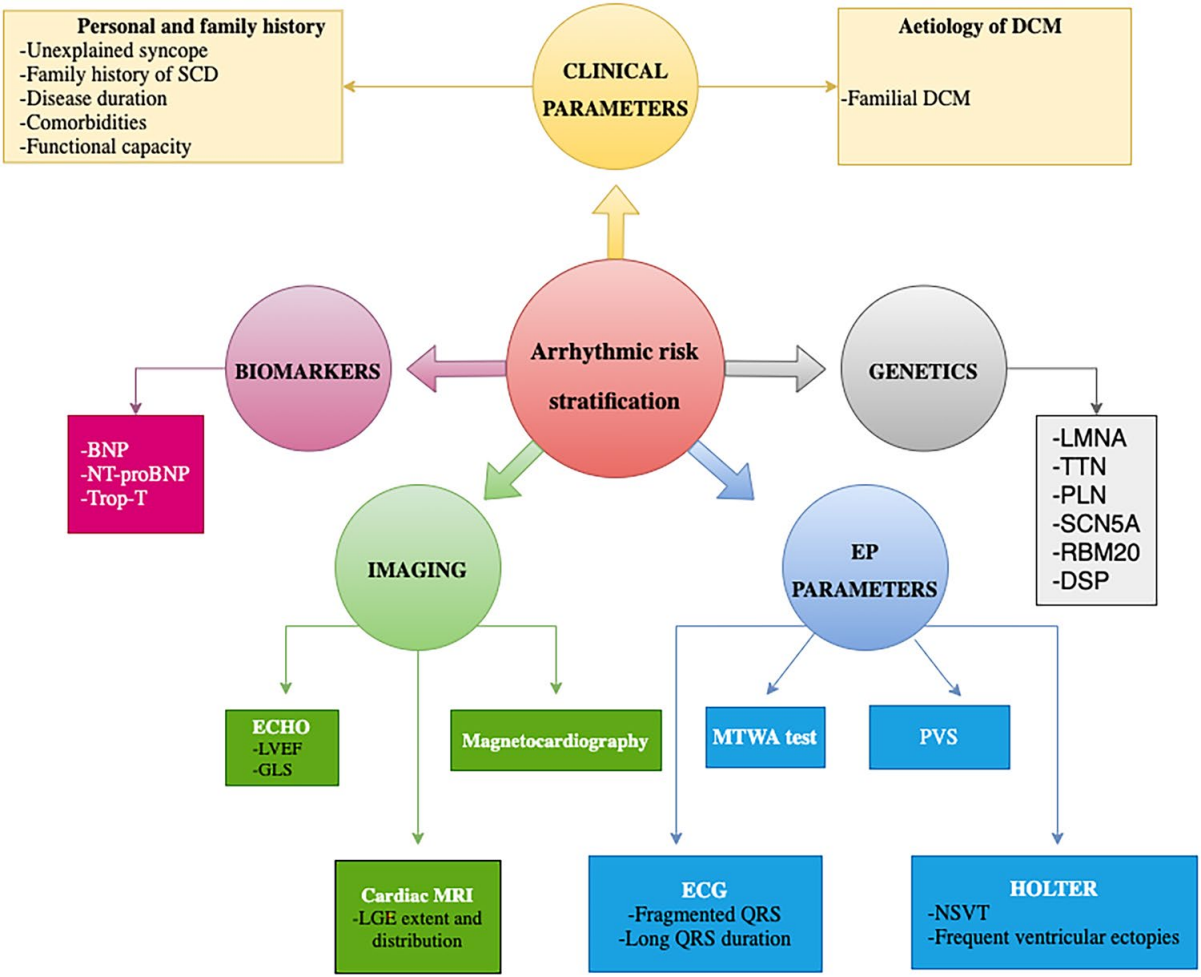
Parameters that have been shown to predict arrhythmic risk in patients with DCM

#### Clinical parameters

##### **Medical history and family history**

Disease duration, unexplained syncope, and family history of SCD are known independent predictors of life-threatening major ventricular arrhythmias (VAs) [19]. In addition, worse NYHA class was found to be independently related to an increased risk of cardiovascular death [20].

##### **Etiology of DCM**

DCM caused by external triggers (acute myocarditis, alcohol, and sustained high-rate supraventricular tachycardia) is associated with a low arrhythmic risk after the removal of causative factor [19], whereas familial DCM associated with certain genes as lamin A/C is associated with higher risk of SCD. Hence, a genuine workup must be performed in all patients with DCM to determine the exact etiology.

#### Electrophysiological parameters

Fragmented QRS (fQRS) and prolonged QRS duration on ECG have been associated with a higher risk of ventricular arrhythmias [21]. In addition, the presence of non-sustained ventricular tachycardia (NSVT) and frequent ventricular ectopy (≥ 1000 premature ventricular contractions or ≥ 50 couplets/24 h) on Holter-ECG was shown to increase the arrhythmic risk in patients with NICM [19].

Microvolt T-wave alternans (MTWA) defined as beat-to-beat changes in repolarisation showed a high sensitivity with a negative predictive value of 97% in the meta-analysis done by Goldberger and his colleagues [21] and hence could potentially be used to exclude patients at low arrhythmic risk despite a severely reduced LVEF. MTWA using the modified moving average technique is currently being investigated by the large observational study, the EUropean Comparative Effectiveness Research to Assess the Use of Primary ProphylacTic Implantable Cardioverter-Defibrillators (EU-CERT-ICD) [22].

Electrophysiological study (EPS) with programmed ventricular stimulation (PVS) might also be useful for identifying patients at higher risk of ICD interventions [23]. However, the absence of standardized EPS protocols hinders the extensive use of PVS for the prognostication of patients with NICM. Currently, ReCONSIDER (arrhythmic risk stratification in nonischemic dilated cardiomyopathy) trial, multicentre, prospective observational trial, is comparing between electrophysiology-driven and CMR-centered approaches for arrhythmic risk stratification in patients with DCM [23].

#### Non-invasive cardiac imaging

##### **Echocardiography**

Echocardiography is the most commonly used imaging technique providing the most important prognostic indicators in patients with DCM, such as LVEF, right ventricular dysfunction, and mitral regurgitation.

##### **Ejection fraction**

The severe reduction of the LVEF (≤ 35%) continues to represent the fundamental criterion on which the choice of ICD implantation in primary prevention is based according to the recommendations of the European Society of Cardiology (ESC) and the American College of Cardiology (ACC). However, using ejection fraction alone as a prediction tool is neither sensitive nor specific for VA and SCD. Only 20–25% of patients with guidelines directed primary prevention ICD receive an appropriate shock within 5 years [8]. In the Maastricht Circulatory Arrest Registry, 68% of patients presenting with SCD had an EF > 30% [63], and in the Oregon Sudden Unexpected Death Study, only one-third of patients presenting with SCD met current criteria for ICD implantation (64).

#### Global longitudinal strain

Speckle tracking–derived left ventricular global longitudinal strain (GLS) has been recently used as useful tool for the identification of subtle LV dysfunction before an overt drop in LVEF with a promising role in SCD risk prediction and arrhythmic risk stratification [24]. However, this role needs to be confirmed in large, randomized trials as compared to conventional methods of arrhythmic risk stratification.

##### **Cardiac magnetic resonance (CMR)**

CMR is recognized as a powerful diagnostic and prognostic tool in NICM. In recent years, late gadolinium enhancement (LGE) imaging has emerged as a strong and consistent tool for prediction of VA and SCD. The most typical pattern seen with LGE is mid wall fibrosis (MWF) which is unique and different from the pattern of fibrosis seen in patients with coronary artery disease (CAD) [25].

**Fig. 2:**
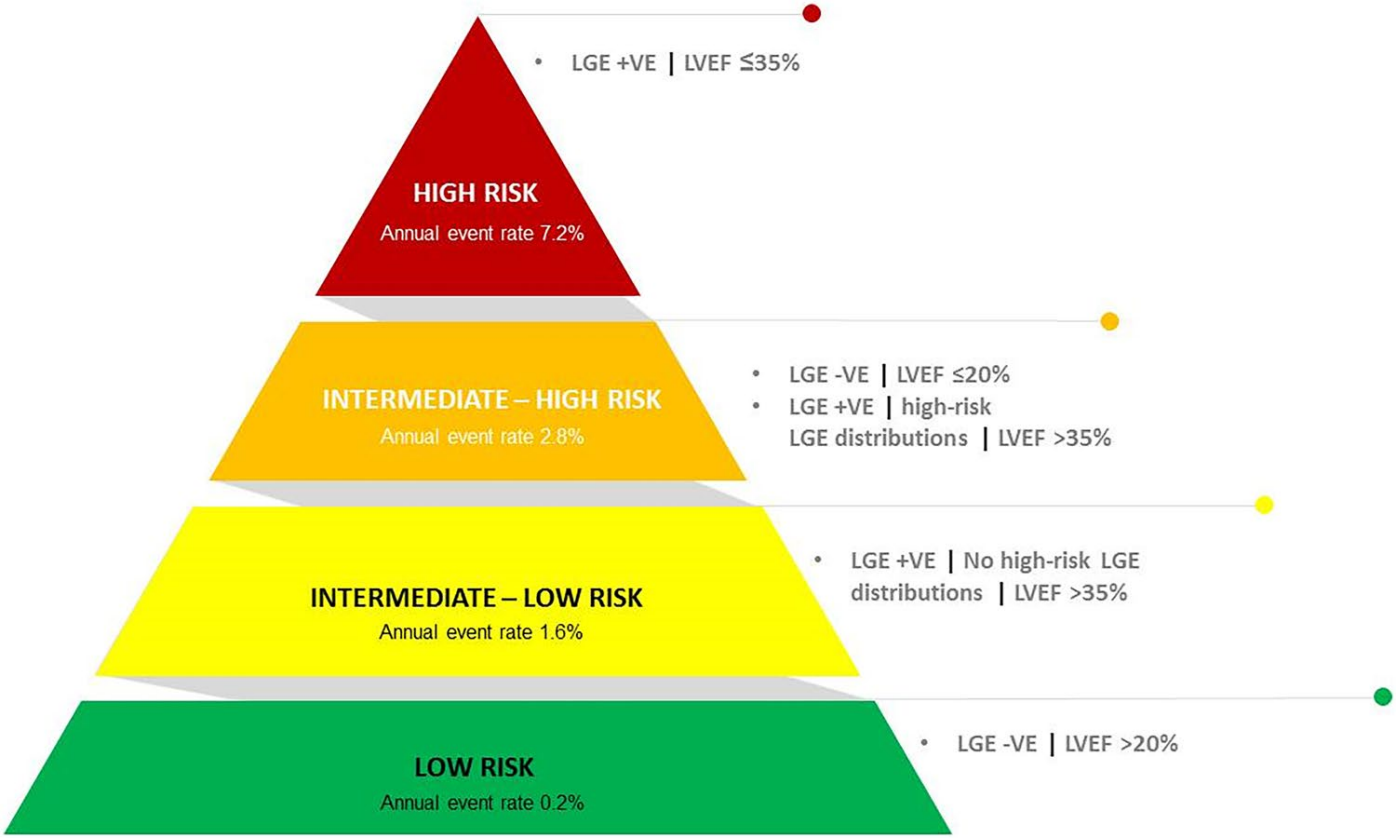
Integrated LVEF- and LGE-based algorithm proposed by Di Marco and his colleagues for arrhythmic risk stratification ^44^. *LGE − *: LGE negative; *LGE* + : LGE-positive. High risk LGE distribution: epicardial LGE, transmural LGE, or combined septal and free-wall LGE

The recently published LGE-DCM study including 1165 patients with DCM found that LGE was an independent and strong predictor of the arrhythmic endpoint with the association being consistent across all strata of LVEF. In addition, patients with high-risk LGE distribution (combined septal and free-wall LGE and those with epicardial or transmural LGE) had also markedly increased arrhythmic risk. A simplified clinical algorithm was derived from this retrospective study combining LGE and 3 LVEF strata (< 21%, 21–35%, > 35%) and was found to be superior to an EF of 35% cutoff only and was able to reclassify the arrhythmic risk of 34% of patients with obvious implications for decision-making on primary prevention ICD [5] (Fig. 2).

All this evidence was the rationale for the currently ongoing CMR-GUIDE (Cardiac Magnetic Resonance GUIDEd Management of Mild-moderate Left Ventricular Systolic Dysfunction) trial which aims to evaluate the efficacy of ICD therapy in patients with ICM or NICM (EF 36 to 50%) and > 2 segments of LGE [26].

##### **Magnetocardiography**

Two small studies showed an association between some MCG findings and major adverse cardiac events and VA [27, 28]. A prospective trial (MAGNETO-SCD; MAGNETO cardiography parameters to predict future sudden cardiac death) will recruit 270 ICM and NICM patients to assess MCG’s prognostic value in SCD [29].

### Biomarkers

Elevated levels of natriuretic peptides have been associated with increased risk of SCD and appropriate ICD therapies, even after adjustment of LVEF and other risk factors [24]. In patients in the DANISH trial with NT-proBNP of < 1177 pg/ml, ICD therapy was associated with lower all-cause mortality but this effect was lost above that cutoff value suggesting that NT-proBNP might have a predictive role in those who are most likely to die from pump failure, and hence will not benefit from primary prevention ICD implantation [30]. A troponin T of > 18 ng/l was also found to be predictive of all-cause mortality in patients with NICM [31].

These biomarkers might have a potential role in facilitating the identification of individuals at increased risk of SCD and VA especially with combining several biomarkers which might improve discrimination especially if used as part of multiparametric algorithm [32, 33].

### Genetics

Over 60 genes have been implicated in NICM, and some are strongly associated with the added risk of fatal arrhythmias [4]. Many variants, however, are associated with incomplete penetrance as well as a complex interaction with environmental triggers, so genetic testing will be better used as an adjunct to other multiparametric measures rather than isolated modality to assess risk of VA and SCD [34].

Several genes have been associated with higher risk of SCD including lamin A/C protein, phospholamban (PLN), sodium voltage-gated channel alpha subunit 5 (SCN5A), RNA binding motif protein 20 (RBM20), filamin C (FLNC), and desmoplakin (DSP) [34, 35].

LMNA gene, which encodes lamin A/C protein, was found to be associated with a high risk of SCD in several observational studies [24]. In a cohort of 269 LMNA mutation positive individuals, NSVT during ambulatory electrocardiographic monitoring, LVEF < 45% at first evaluation, male sex, and nonmissense mutations were independent risk factors for VA with malignant VA being observed only in persons with ≥ 2 of these risk factors [24].

Titin (TTN) is the commonest gene mutation in patients with DCM, being observed in up to 25% of familial and 15% of sporadic cases of NICM [33]. The presence of TTN as a risk predictor of SCD is still unclear as some studies showed that TTN is independently associated with LV reverse remodeling and low risk of heart failure hospitalization and cardiovascular death [36], while other studies found that having a TTN-truncating variants (TTNtv) was associated with a higher risk of receiving appropriate ICD therapy (shock or antitachycardia pacing) for VT or VF [37].

## Left ventricular reverse remodeling (LVRR)

LVRR is a well-established predictor of a good prognosis in patients with DCM and can occur in up to 40% of cases [38]. LVRR is usually defined as an absolute increment in LVEF of 10% or more reaching a final value of 35% or more associated with reduction of 10% or more in left ventricular end-diastolic dimension (LVEDD) within 6 to 18 months from DCM diagnosis [39, 40]. Patients with LVRR have good prognosis, with transplant-free survival of 95% versus 71% in those who do not at 180-day follow-up [41].

The ESC/ACC guidelines recommend primary prevention ICD in patients with DCM, symptomatic heart failure (NYHA class II–III), or an LVEF ≤ 35% despite ≥ 3 months of treatment with optimal medical therapy [42, 43]. However, LVRR starts in 6 months and continues for 2 years from initiation of therapy with relatively lower risk of ventricular arrhythmias in patients with LVRR [44]. Therefore, longer waiting period should be considered in patients with high likelihood of LVRR before ICD implantation and a LVRR predicting score may be helpful when making these therapeutic decisions.

### Predictors of LVRR

#### Etiology of heart failure

The LVRR rate is directly related to the etiology of heart failure. Patients with ICM have a lower rate of LVRR, whereas patients with inflammatory DCM and post myocarditis DCM [45] have relatively high LVRR rate after spontaneous viral elimination and subsequent downregulation of intramyocardial inflammation [46, 60–62].

#### Blood pressure

Hypertension (HTN) at baseline was also reported to be a predictor of LVRR in patients with DCM in multiple studies [21, 47, 48] especially that anti-failure medications with proven mortality benefit can be used to modulate the afterload and can be relatively easily titrated in patients with HTN as compared to patients with low baseline blood pressure.

#### Symptom duration

Prolonged symptom duration could lead to irreversible myocardial damage. Symptom duration of more than 3 months has been associated with lower rate of LVRR. Hence, early diagnosis and treatment are important for LVRR in patients with DCM [40].

#### LVEF

Low LVEF has been found to be an independent predictor of LVRR in more than one study [47, 48] and a significant relationship between LVEF and genetic variants has been confirmed [49]. TTN variants are independently associated with low EF at baseline and LVRR. In contrast, patients with LMNA mutations showed high baseline LVEF and no LVRR [49]. Hence, combining LVEF with genetic testing can help to predict LVRR even if the LVEF is substantially reduced.

#### Family history of DCM

The presence of a family history of DCM was found to be associated with more myocardial fibrosis detected by LGE and higher incidence of cardiac events as compared to patients without a family history of DCM [50].

#### QRS duration

A wide QRS indicates intraventricular conduction defect and LV dysfunction and is associated with diffuse myocardial fibrosis, arrhythmia, and mortality. Patients with left bundle branch block and longer QRS duration are associated with a low probability of left ventricular reverse remodeling and should be considered for early CRT implantation [40].

A simplified LVRR predicting score using five predictors has been proposed including hypertension (1 point), no family history of DCM (2 points), symptom duration < 90 days (1 point), LVEF < 35% (2 points), and QRS duration < 116 ms (1 point) and the total score is the sum of the predictors. The LVRR predicting score could help to stratify the LVRR rate, and the score of > 5 was found to be an independent predictor of presence of LGE on CMR [40].

### Other factors affecting primary prevention ICD implantation decision-making

#### Age and life expectancy

The benefit of the ICD is governed by the balance between the risk of SCD and the risk of death from other causes, as well as comorbidities. In addition, advanced age has been shown to be an independent risk factor associated with complications at the time of ICD implantation and with no significant impact on these patients’ quality of life. Hence, it is particularly important to consider general predictors of ICD effectiveness such as age, comorbidities, and frailty associated with a mortality risk that competes with sudden arrhythmic death and shared decision‐making is highly recommended in these cases.

#### End stage renal disease

Patients with end-stage renal disease (ESRD) especially those requiring renal replacement therapy have a 14-fold increased risk of death compared with the general population, with the largest category of cause-specific death believed to be attributable to cardiovascular disease [51]. In the prospective, randomized, controlled ICD2 trial including well-screened and well-treated population undergoing dialysis, prophylactic ICD therapy did not reduce the rate of SCD or all-cause mortality which remained high [51]. In addition, patients with ESRD were excluded from the pivotal trials that established the mortality benefit of the ICDs in these high-risk patients. Hence, the benefit of ICD therapy in patients on dialysis who meet these guideline indications is still unclear [51].

## Role of medical therapy in prevention of SCD

The use of optimal medical therapy leads to reduction of mortality in HF patients including sudden cardiac death [52]. Beta-blockers (BBs), mineralocorticoid receptor antagonists, and ARNI significantly reduce SCD and improve overall survival among individuals with HF and reduced ejection fraction. However, there was no evidence of effectiveness of ARBs to reduce neither all-cause mortality nor SCD and ACEIs to reduce SCD events [53].

According to the most recent ESC and ACC guidelines for the diagnosis and treatment of acute and chronic heart failure published in 2021 and 2022, respectively, optimal medical therapy (OMT) for patients with HFrEF should ideally include the use of Class I recommended drugs for HFrEF including ACE-I/ARNI, BBs, MRAs, and SGLT2-I [42, 43]. However, the ICD trials predate the use of ARNI and SGLT2-I inhibitors and whether the early simultaneous start of the four lines of treatment can delay or affect the timing and the need of primary prevention ICD implantation is still unclear.

## Optimal timing of primary prevention ICD implantation

The optimal timing of ICD implantation in dilated NICM is still not well-established. The waiting period of 3 months mentioned by ESC and ACC HF guidelines before implantation of ICD is mainly based on expert opinion [42, 54] and there are multiple caveats to this waiting period. Firstly, reverse remodeling that is augmented by pharmacological therapy often extends beyond 3 months. Effects of ACEIs and ARBs on EF were observed after a mean duration of 6 months [55] and the use of ARNI was associated with improvement of cardiac reverse remodeling and LVEF beyond 3 months follow-up [56]. Another caveat is that during those 3 months, the patient is not protected by ICD against SCD and up titrating the medical treatment is lengthy and may take time beyond 3 months with most of the patients still taking their initial prescribed doses of medial therapy [57] and even after 1 year of follow-up, many patients may not be on target doses of optimal medical therapy [58].

New algorithm for rapid sequencing of the ACEI/ARNI, BB, SGLT2i, and MRA in 3 steps with a timeframe of 4 weeks has been proposed which can help to keep the patients on the guideline-directed medical therapy (GDMT) in short time to get maximum benefit of the pharmacological treatment of heart failure, reduce the mortality and risk of SCD, and increase the chance of LVRR [59]. In addition, simplified scoring system for prediction of LVRR could help to identify low-risk patients with DCM whom would benefit from longer waiting period and avoid unnecessary primary prevention ICD implantation [40]. On the other hand, early ICD implantation should be considered in patients with high-risk features as positive LGE with high-risk LGE distributions on CMR [5] or confirmed genetic mutation associated with higher risk of SCD as lamin A/C protein, PLN, SCN5A, RBM20, FLNC, and DSP [60–62].

In conclusion, proper arrhythmic risk stratification and LVRR prediction through multiparametric approach are crucial to identify high-risk patients who will benefit from early ICD implantation and low-risk patients whom longer waiting period seems reasonable especially in the presence of multiple LVRR positive predictors.

## Cost-effectiveness of primary prevention ICD implantation

Compared to medical therapy only, single lead ICD was found to be cost-effective in primary prevention of sudden cardiac death in both ischemic and non-ischemic left ventricular dysfunction based on systematic review of multiple RCTs including DANISH trial [63] with estimated incremental cost-effectiveness ratio (ICER) being higher in non-ischemic compared to ischemic patients and slightly less in NICM patients aged less than 68 years. Another cost-effectiveness analysis of implantable cardiac devices was conducted using pooled individual patient data from 13 RCTs and ICD was cost-effective in NYHA I–III patients with QRS < 120 ms and non LBBB morphology compared to medical therapy alone and the result was consistent in NICM subgroup [63].

## Current guideline recommendation for primary prevention ICD implantation

In the 2021 ESC HF guidelines, the recommendation for primary prevention ICD implantation in patients with NICM was downgraded from class I to class IIa [43] based on the results of the DANISH trial [12] with ICD implantation to be considered only in symptomatic patients (NYHA class II–III), with LVEF ≤ 35% despite ≥ 3 months of OMT, provided they are expected to survive longer than 1 year with good functional status (Class of Recommendation: COR IIa, Level of Evidence: LOE A) [43]. In addition, early primary prevention ICD implantation should be considered in patients with DCM and a confirmed LMNA, RBM20, PLN, and FLN mutation guided by risk factors (NSVT during ambulatory ECG monitoring, LVEF < 45% at first evaluation, male sex, and non-missense mutations (insertion, deletion, truncations, or mutations affecting splicing)) [43]. Finally, ICD therapy was not recommended in patients in NYHA class IV, with severe symptoms refractory to pharmacological therapy, unless they are candidates for CRT, a ventricular assist device (VAD), or cardiac transplantation (COR III, LOE C) [43].

Similarly, the ACC Guidelines for Management of Patients with HF recently published in 2022 recommended ICD implantation in patients with NICM with NYHA class II–III and LVEF of ≤ 35%, despite GDMT, if meaningful survival of greater than 1 year is expected (COR I, LOE A) [42]. In addition, implantation of ICD was reasonable to decrease sudden death in patients with genetic arrhythmogenic cardiomyopathy (LMNA/C, desmosomal proteins, phospholamban, or filamin-C) with high-risk features of sudden death and EF ≤ 45% (COR IIa, LOE B-NR) [42], especially in patients with NICM due to a lamin A/C mutation who have 2 or more risk factors (NSVT, LVEF ≤ 45%, nonmissense mutation, and male sex), if meaningful survival of greater than 1 year is expected according to the ACC Guideline for Management of Patients with VAs and the Prevention of SCD published in 2017 (COR IIa, LOE B-NR) [61]. Finally, in patients with medication-refractory NYHA class IV HF who are not also candidates for cardiac transplantation, a left ventricular assist device (LVAD), or a CRT defibrillator that incorporates both pacing and defibrillation capabilities, an ICD should not be implanted (Class III, LOE C-EO) [42, 61].

The current ESC and ACC recommendations for primary prevention ICD therapy are summarized in Table 2.

**Table 2:** Comparison of latest European and American Guidelines for primary prevention ICD therapy recommendation

**NYHA class**	**ESC**	**ACC**
**LVEF ≤ 35% + NYHA class I despite 3 months of OMT**	-	-
**LVEF ≤ 35% + NYHA class II–III despite 3 months of OMT**	IIa	I
**LVEF ≤ 35% + NYHA class IV despite 3 months of OMT**	III	III

## Multiparametric integrated approach

In conclusion, integrating all the above parameters is crucial with individualized management of DCM patients based on proper arrhythmic risk stratification and proper LVRR prediction for proper selection of DCM patients who will benefit from primary prevention ICD and proper timing of the ICD implantation and hence improving the outcomes.

**Fig. 3:**
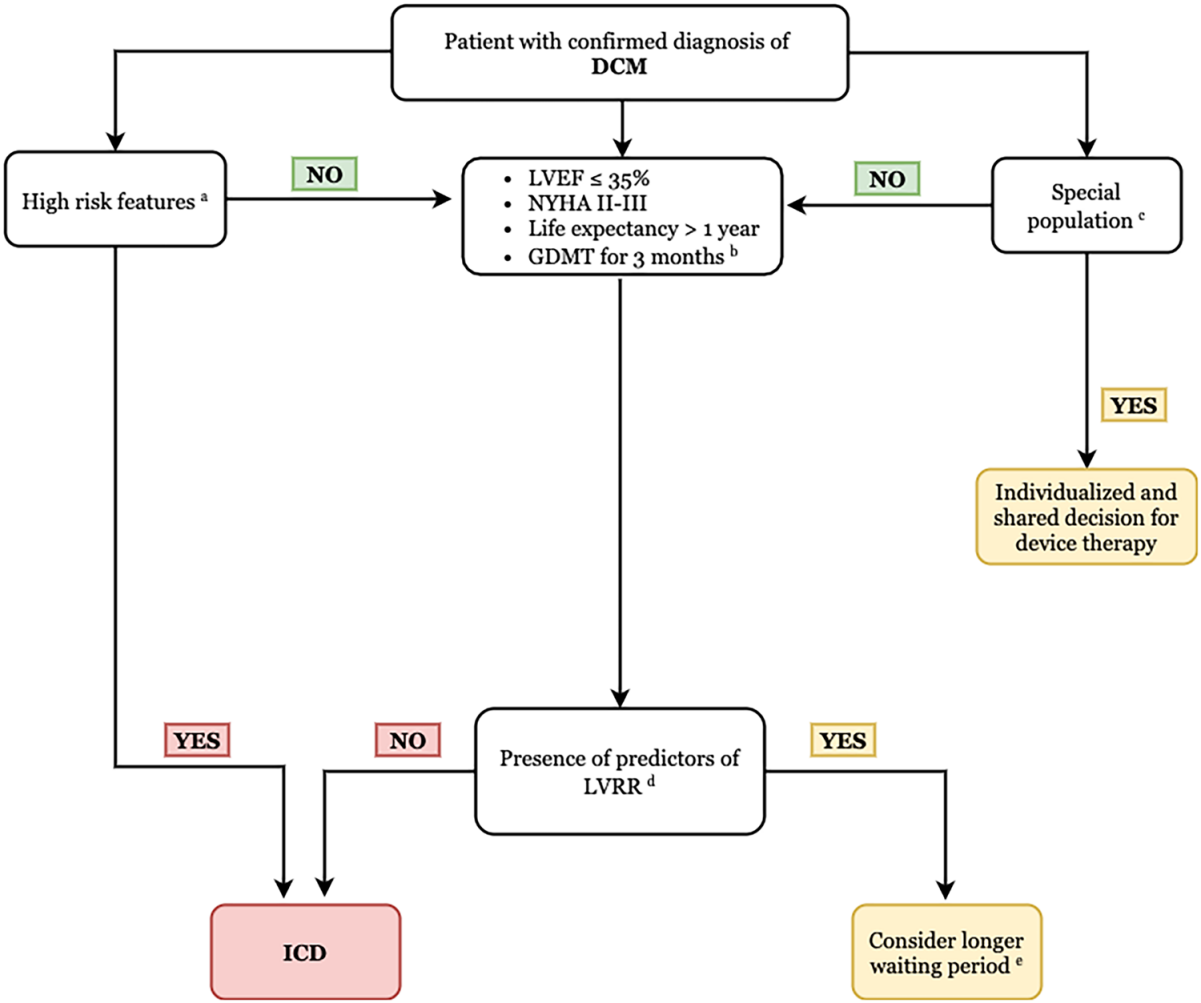
Proposed algorithm for timing and patient selection for primary prevention ICD implantation among patients with DCM. **a** Special population: advanced renal dysfunction/dialysis, elderly/pediatric, class I/IV heart failure, and adult congenital heart. **b** GDMT: ACEI/ARNI, BB, MRA, and SGLT2i. **c** High-risk features: positive LGE with high-risk LGE distributions on CMR, confirmed LMNA, RBM20, PLN, and FLN mutation with two or more risk factors (NSVT during ambulatory ECG monitoring, LVEF < 45% at first evaluation, male sex, and non-missense mutations (insertion, deletion, truncations, or mutations affecting splicing)). **d** Predictors of LVRR: narrow QRS complex, absence of LGE, hypertension, absence of FH of DCM, absence of symptoms or symptom duration of less than 3 months, and absence of LMNA gene variants. **e** Regular follow-up (clinical + ECG + Holter + Echo) is recommended during this waiting period

We proposed this multiparametric integrated approach to guide patient selection and timing of ICD implantation to accurately identify DCM patients who will benefit from primary prevention ICD implantation and avoid unnecessary ICD implantation (Fig. 3). However, randomized controlled studies are still needed to evaluate the outcomes of primary prevention ICD implantation in DCM patients based on this multiparametric integrated approach.

## Abbreviations

ACC: American College of Cardiology
ACEI: Angiotensin converting enzyme inhibitor
AF: Atrial fibrillation
AHA: American Heart Association
AMIOVIRT: Amiodarone versus implantable cardioverter-defibrillator randomized trial
ARB: Angiotensin receptor blocker
ARNI: Angiotensin receptor neprilysin inhibitor
BB: Beta blocker
BNP: B-type natriuretic peptide
CAD: Coronary artery disease
CAT: Cardiomyopathy trial
CMR: Cardiac magnetic resonance
CMR-GUIDE: Cardiac Magnetic Resonance GUIDEd Management Of Mild-Moderate Left Ventricular Systolic Dysfunction
COMPANION: Comparison of medical therapy, pacing and defibrillation in heart failure
COR: Class of recommendation
CRT: Cardiac resynchronisation therapy
CRT-D: Cardiac resynchronisation therapy – defibrillator
CRT-P: Cardiac resynchronisation therapy – pacemaker
CV: Cardiovascular
DANISH: Defibrillator implantation in patients with non-ischemic systolic heart failure
DCM: Dilated cardiomyopathy
DEFINITE: Defibrillators in non-ischemic cardiomyopathy treatment evaluation
DSP: Desmoplakin
ECG: Electrocardiogram
EF: Ejection fraction
EPS: Electrophysiological study
ESC: European Society of Cardiology
ESRD: End-stage renal disease
FLN: Filamin
fQRS: Fractionated QRS
GDMT: Guideline-directed medical therapy
GLS: Global longitudinal strain
GO-DCM: Defining the genetics, biomarkers and outcomes for dilated cardiomyopathy
HF: Heart failure
HFrEF: Heart failure with reduced ejection fraction
HR: Hazard ratio
HRS: Heart Rhythm Society
HTN: Hypertension
ICD: Implantable cardioverter defibrillator
ICM: Ischemic cardiomyopathy
LGE: Late gadolinium enhancement
LOE: Level of evidence
LV: Left ventricle
LVEDD: Left ventricular end-diastolic dimension
LVEF: Left ventricular ejection fraction
LVRR: Left ventricular reverse remodeling
MADIT-II: Multicentre Automatic Defibrillator Implantation Trial
MAGNETO-SCD: MAGNETO cardiography parameters to predict future sudden cardiac death
MCG: Magnetocardiography
MRA: Mineralocorticoid receptor antagonist
MTWA: Microvolt T-wave alternans
MVA: Monomorphic ventricular arrhythmia
MWF: Mid wall fibrosis
NICM: Non-ischemic cardiomyopathy
NSVT: Nonsustained ventricular tachycardia
NT-ProBNP: N-terminal pro B-type natriuretic peptide
NYHA: New York Hart Association
OMT: Optimal medical therapy
PLN: Phospholamban
PVCs: Premature ventricular contractions
PVS: Programmed ventricular stimulation
RBM20: RNA-bonding motif protein 20
RCT: Randomized controlled trial
ReCONSIDER: Arrhythmic risk stratification in non-ischemic dilated cardiomyopathy
SCA: Sudden cardiac arrest
SCD: Sudden cardiac death
SCD-HeFT: Sudden cardiac death in heart failure trial
SCN5A: Sodium voltage-gated channel alpha subunit 5
SGLT2-I: Sodium glucose transporter 2 inhibitor
TTN: Titin
TTNtv: Titin truncating variant
VA: Ventricular arrhythmia
VAD: Ventricular assist device
VF: Ventricular fibrillation
VT: Ventricular tachycardia.
